# Confined Reactivity in the van der Waals Gap beneath Graphene: Supply-Limited Kinetics and Emergent Reaction Pathways

**DOI:** 10.1021/acsnano.5c12130

**Published:** 2026-03-04

**Authors:** Hossein Mirdamadi, Rui Wang, Jiří David, Tianle Jiang, Yanming Wang, Karel Vařeka, Michal Dymáček, Petr Bábor, Tomáš Šikola, Miroslav Kolíbal

**Affiliations:** † CEITEC BUT, 48274 Brno University of Technology, Purkyňova 123, Brno 612 00, Czech Republic; ‡ School of Mechanical Engineering, 12474 Shanghai Jiao Tong University, 800 Dongchuan Rd, Shanghai 200240, China; § Institute of Physical Engineering, 48274 Brno University of Technology, Technická 2, Brno 616 69, Czech Republic; ∥ University of Michigan-Shanghai Jiao Tong University Joint Institute, 12474 Shanghai Jiao Tong University, 800 Dongchuan Rd, Shanghai 200240, China; ⊥ Global Institute of Future Technology, 12474 Shanghai Jiao Tong University, 800 Dongchuan Rd, Shanghai 200240, China

**Keywords:** in situ scanning electron microscopy, etching, graphene, intercalation, O_2_, H_2_, CO

## Abstract

The confinement of molecules within the van der Waals (vdW) gap between a two-dimensional (2D) material and a catalytic substrate offers a promising route toward the development of molecule-selective catalysts with increased reaction rates and access to chemically distinct reaction environments. However, identifying the kinetic limitations and mechanistic consequences of such confined reactions remains challenging. Here, we employ an inverted wedding cake configuration of multilayer graphene on platinum to study the dynamics of graphene etching within the vdW gap by O_2_, H_2_, and CO using in situ scanning electron microscopy. Under the experimental conditions explored (up to *p* = 1.4 × 10^–2^ Pa and *T* = 1000 °C), the etching reactions are supply limited for O_2_ and H_2_. The reaction-limited regime is not observed even for CO, despite its anomalously enhanced transport resulting from a pronounced lifting of the vdW gap. Reactive molecular dynamics simulations reveal that confinement within the vdW gap enables additional CO-mediated etching pathways that are absent on open Pt surfaces. Our results demonstrate that intercalation does not primarily reduce reaction barriers but instead creates a confined, high-chemical-potential nanoreactor in which new reaction pathways can be accessed at comparatively low external pressures.

## Graphical abstract

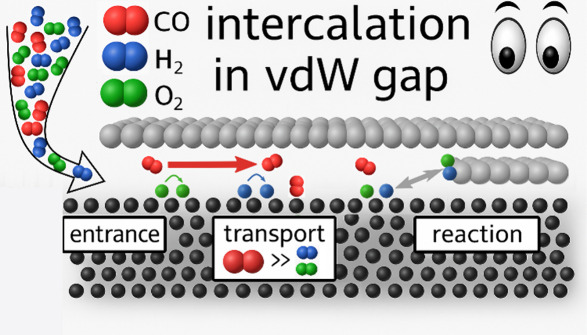

## Introduction

Intercalation is the insertion of foreign atoms, molecules, and ions within the matrix of layered materials. The most successful technology that builds on this phenomenon is the lithium-ion battery, derived from the initial research of Stanley M. Whittingham, focused on the intercalation of lithium ions within layered TiS_2_.^1^ In follow-up studies, graphite and oxide electrodes have been introduced,^2–5^ facilitating the rapid developments of lithium battery technology. Recently, with renewed interest in 2D materials, the intercalation of foreign atoms in the van der Waals (vdW) space between individual layers and the substrate has been utilized for many purposes.^6^ After intercalation, the distance between the layers may increase; an intercalated crystal could be utilized for gas storage^6^ and the intercalated layers are easier to peel off due to weakened vdW bonding.^7^ In the case of monolayer 2D materials positioned on the substrate surface, weakening of the interaction between the 2D material and the substrate due to intercalation may uncover the inherent electronic structure of the 2D material,^6,8^ bringing the original promise of unrivaled electronic properties and applications in electronic devices back on stage.^9^ Moreover, intercalated 2D materials have been reported to exhibit properties unseen in their free-standing counterparts.^10,11^

A mechanistic understanding of the initiation of the intercalation process has been established by utilizing graphene on a metal support as a prototypical example. In the absence of on-surface molecular or atomic species, the graphene edges are bonded to the metal atoms, effectively sealing the space between the metal and graphene.^12,13^ The intercalation is possible only after the edges become terminated by adsorbates and “lifted” up, thus opening the van der Waals space for diffusing atoms and molecules^13,14^ It has been previously shown that this process is viable even at very low temperatures,^13,15,16^ and, thus, a prolonged exposure of a graphene-covered metal to the atmosphere may result in intercalation.^17^

While many researchers focus on the properties of 2D materials after intercalation, it has been shown that the vdW gap between the substrate and a 2D material cover may facilitate unique material synthesis or drive chemical reactions.^18,19^ The formation of new 2D materials within the gap^20^ has been demonstrated for 2D GaN,^21^ air-sensitive monolayer indium,^22,23^ gallium,^24,25^ bismuth,^26^ and even 2D gold.^27^ Another appealing research direction is performing chemical reactions within the van der Waals gap,^28–35^ in particular gas-phase heterogeneous catalysis.^36^ Several recent reports suggest that within the gap, the catalytic activity of the substrate that drives the reaction is increased.^33^ The mechanism behind, generally called the “confinement effect”, is still not fully explored. The possible explanations include deviations from the bulk thermodynamic limits in nanoscale systems^37^ or lowered activation energies of related temperature-activated processes^38,39^ via charge transfer from a 2D monolayer.^40^ Theoretical modeling suggests a significantly increased pressure within the gap,^19,28^ which may affect molecular shapes^41^ and, hence, the reaction rate. The rate enhancement can also come from the third species present during the reaction, facilitating the entrance of the van der Waals gap for larger molecules involved in the reaction of interest.^42^

Going one step further, a full understanding of the chemical reaction kinetics requires knowledge of limiting processes, reaction rates, diffusion coefficients, etc. Obtaining these data from experiments is very challenging, because monitoring of the intercalated species requires utilization of surface sensitive techniques under *operando* conditions. Up to date, mostly X-ray photoelectron spectroscopy (XPS) and scanning tunneling microscopy (STM) have been jointly used to identify and monitor the intercalated species,^13,43,44^ in few cases corroborated by low-energy electron microscopy (LEEM).^40,45^ Direct microscopic observations of diffusion are possible via transmission electron microscopy^19^; yet, these experiments are limited to a specific case of atomic motion within 2D material stacks. Quantitative experimental data on atomic or molecular dynamics in the van der Waals gap between a metal and a 2D material are missing. Only recently such data have been reported via indirect, yet ingenious experimental observations.^14,39,42,46–49^

Here, we study O_2_, H_2_, and CO intercalation under graphene on the platinum surface due to their principal importance in fundamental catalytic reactions (e.g., CO oxidation over platinum). First, we characterize the intercalated superstructures by microdiffraction experiments in LEEM together with reflectivity measurements. Next, we utilized in situ scanning electron microscopy and monitored the etching of multilayer graphene in an inverse wedding cake configuration in real time. We show that the etch rate under the graphene is mass supply limited (within the pressure range used here, i.e., up to 1.4 × 10^–2^ Pa) and, for all three gas molecules of interest, that the etching rate of the buried graphene layer is smaller compared to the etching rate of the overlayer graphene. Moreover, we have derived the activation energies for diffusion and compared them to the diffusion on the free surface.^50^ Additionally, molecular dynamics simulations suggest different alternative pathways for graphene etching by CO, which are exclusive to the van der Waals space and are not observed on the pristine platinum surface.

## Results

### Graphene Intercalation

Direct microscopic observation of intercalation is quite challenging. Instead, one can utilize the secondary effects caused by intercalation to indirectly monitor the process. X-ray photoelectron spectroscopy (XPS) is commonly used to monitor intercalation of 2D materials, because the change in interlayer coupling after intercalation results in different binding energies.^13,43^ However, because of weak coupling of graphene to Pt, the shifts of the relevant binding energies are too small to provide a conclusive proof of intercalation in our experiments (see Figure S1, Supporting Information). As an alternative to XPS, observation of moiré pattern fading has been employed before.^45,51^ Moiré effects appear in both scanning tunneling microscopy^32^ as well as in electron diffraction.^45,51^ Hence, in order to get detailed insight into the intercalation process, we have employed the measurement of electron reflectivity (Figure 1e) in a low-energy electron microscope (LEEM) in addition to low-energy electron diffraction (Figure 1a,d) on a single crystal (111) platinum partially covered with monolayer graphene (see the scanning electron microscope (SEM) image in Figure 1b). The appearance of several graphene diffraction spots as well as two moiré patterns (Figure 1a, right-half) reflects the polycrystalline nature of the studied graphene layers. Yet, it is worth noting that similar to other reports,^13^ we were not able to intercalate graphene if a full monolayer was prepared on the metal surface, suggesting that solely the graphene edges act as the entrance sites for intercalation (Figure 1c) under the pressure conditions studied here.

**Figure 1:**
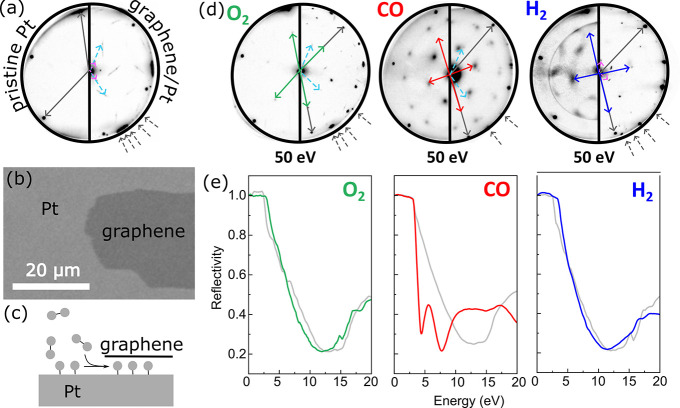
Intercalation of different molecules under the graphene, documented by low-energy electron diffraction (LEED, panel d) and reflectivity of electrons (e). (a) Low-energy electron diffraction patterns (50 eV), showing pristine Pt(111) (left) and graphene-covered Pt(111) (right); see schematic at the bottom. Several of the many graphene-related spots at the circumference of the pattern are marked with dashed gray arrows. The other arrows depict the unit vectors of the respective unit cells: Pt(111) (gray), graphene moiré demonstrated by the spots around the central (0,0) spot (pink) and by the $\left(\sqrt{7}\times \sqrt{7}\right)R19.1^{{}^{\circ}}$ superstructure (light blue). (b) SEM image of the monolayer graphene flake. (c) Schematic of the intercalation process. (d) LEED patterns taken at Pt(111) (left-half circles) and graphene-covered platinum (right-half circle) after exposure to O_2_, CO, and H_2_ at room temperature. CO diffraction patterns were obtained as a composition of diffraction patterns taken at energies of 10–50 eV. The arrows represent the unit vectors of the respective unit cells of adsorbed (left-halfcircles) and intercalated (right-half-circles) superstructures as well as the preserved moiré patterns (right-half circles, dashed arrows). Superstructures formed on graphene-covered platinum (right-half circles and solid arrows) appeared after a prolonged period compared to those on graphene-free platinum. These times differ for each molecule (see the text for details). (e) Reflectivity-energy curves acquired by sweeping the incident energy of electrons in LEEM. Gray curves: platinum covered with graphene under vacuum conditions; colored curves: platinum covered with graphene after exposure to the respective gas (1–2 × 10^–6^ Pa) at different times (up to 90 min for O_2_, 10–20 min for CO, 20–30 min for H_2_) at room temperature.

First, exposure of the sample to different molecular gases was performed in LEEM (see Methods) at room temperature. The diffractograms acquired using the microdiffraction aperture on pristine Pt changed quickly in response to gas exposure (within 2–3 min), reflecting the formation of (2 × 2)-O superstructure after O_2_ exposure, while both CO and H_2_ induce c(4 × 2) superstructure, in agreement with previous reports (O_2,_ ^52^ CO^53,54^ ). At areas covered with graphene, the appearance of new spots was significantly delayed (up to 90 min for O_2_, 10–20 min for CO, and 20–30 min for H_2_). In all cases, the gas-induced superstructures were the same at pristine and graphene-covered platinum, being the first evidence of intercalation. Hence, the confinement of the adsorbate molecules in the van der Waals space does not affect preferred adsorption sites.^13,44^ An important observation is that the moiré-related spots, which are visible on graphene-covered areas (see Figure 1d, left-half), do not disappear after intercalation and are still present in the diffractograms, although with a lower intensity.

Reflection of low-energy electrons from the surface is a very sensitive measure of any surface modification.^55^ The energy dependence of the reflectivity measured in graphene-covered areas shows an abrupt characteristic decrease at around 3–5 eV (the work function of pristine Pt is ∼5.8 eV),^56^ profound minimum between 12 and 15 eV, followed by a local maximum peaking at 20 eV (Figure 1d). Intercalation of H_2_ and O_2_ results in a very small shift of the reflectivity minimum toward lower energies. In contrast, CO intercalation induces remarkable changes in the reflectivity: the shift to the lower energies is significantly larger and an additional minimum shows up. It has been demonstrated previously that the minimum in the reflectivity shifts toward lower energies because of the increase of the van der Waals gap height, and even additional minima may appear.^57^ Hence, contrary to O_2_ and H_2_ intercalation, our reflectivity data suggest that the CO intercalation significantly increases the vdW gap height. Importantly, the gap remains lifted even at elevated temperatures (see SI, Figure S2) while, at the same time, the adsorbate-induced diffraction patterns from below graphene disappear. This observation is important for deducing the mass-transport mechanism of intercalated molecules, which is discussed later.

### Kinetics of Buried Graphene Etching in the vdW Gap

We have followed the kinetics of intercalated molecules in the van der Waals gap indirectly by monitoring the etching of the buried graphene layers of multilayer graphene. First, we have prepared multilayer graphene in an inverted weeding cake configuration, consisting of an overlayer and 1–2 buried graphene layers, further denoted as second and third buried layers, respectively (schematically shown in Figure 2a, see Methods for description). Still at elevated temperature, we introduced a precursor for etching (O_2_, H_2_, or CO) while monitoring the multilayer flakes in real time with an electron beam. It should be noted that specifically for CO, the graphene etching is observed for partial CO pressures in the 10^–3^ Pa range; at lower pressures, graphene is not etched in the CO environment (Figure S3). The experimental conditions (up to *p* = 1.4 × 10^–2^ Pa and *T* = 1000 °C) were chosen to avoid etching outbursts within the basal plane of the graphene overlayer; instead, we managed to form an etch front that moves across a sample in one direction (see Figure 2b, the orange arrows, for an example of graphene etching by oxygen captured by in situ SEM). As the etch front advances, the buried graphene layers start to etch as well. Note that the etching of the buried layers starts even before the overlayer etch front reaches their edges, but it occurs only at a certain etch front-edge distance. In the following text, we will call the distance between the overlayer etch front and the buried graphene edge the ‘mean critical distance’, ⟨*d*_c_⟩, since it is apparent that the etching is initiated by the precursor molecules that diffused toward the buried graphene layers within the van der Waals gap after intercalation under the overlayer graphene. This observation serves as another fingerprint of the etchant intercalation beneath the graphene overlayer.

**Figure 2:**
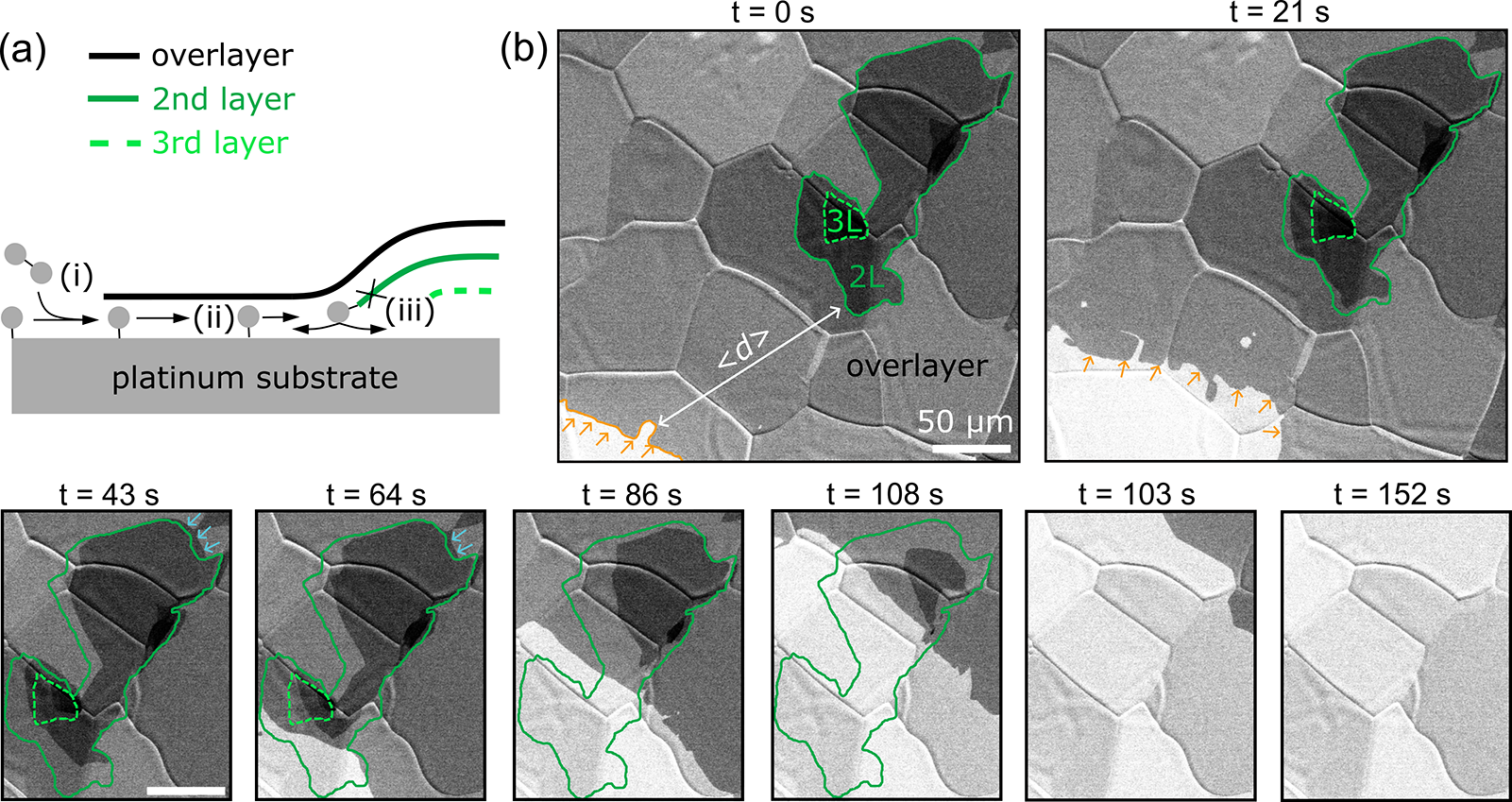
Image sequence of multilayer graphene etching by oxygen observed in situ in SEM (5 keV and 3 nA electron beam). (a) A schematic of the intercalation process in the multilayer graphene stack (black, overlayer; light green, green, buried layers), denoting the possible limiting processes: (i) entrance to the vdW gap, (ii) mass-transport within the vdW gap, and (iii) etching reaction, followed by mass-transport of reaction products. (b) The etching front of the top layer moves toward the multilayer region. The etching front is marked with an orange line, and the direction of the etching front movement is marked with orange arrows. Both the second and third buried graphene layers (encircled by dashed and full green lines, respectively) start to be etched even before the overlayer etch front reaches their edges. Additionally, graphene growth was observed on the opposite sides of the flakes for a short period (blue arrows). Oxygen pressure was 6.6 × 10^–4^ Pa, temperature 1000 °C.

The electron beam enables visualization of the reaction and monitoring its progress in real time and direct space, while having a negligible effect on the reaction rate (see Figure S4, Supporting Information). This allows for a quantitative evaluation of the scaling behavior of the etching kinetics, which in turn enables identification of the rate-limiting mechanism. Etching of graphene requires, at a minimum, the following three processes: (i) supply of intercalated molecules through graphene edges, (ii) transport of the intercalated molecules to the graphene domain, and (iii) attachment and/or reaction with carbon atoms at the graphene domain edges, followed by removal of reaction products. In the reaction-limited regime (iii), the areal etch rate $\frac{dA}{dt}$ scales with the flake perimeter. Consequently, the flake radius decreases linearly with time ( $r=\sqrt{A}=r_{0}-\varepsilon .t$ , where ε is the (constant) etch rate and *r*_0_ is the initial flake radius). In contrast, when the reaction is limited by the availability of etchant molecules (the so-called supply-limited regime, encompassing processes (i) and (ii)), $\frac{dA}{dt}$ is constant and the reaction kinetics are nonlinear, yielding *r* = *r*_0_ – ε. *t* ^2^. In Figure 3a, we plot the evolution of the square root of the flake area as a function of time for the second and third buried graphene layers during etching by oxygen. Both dependencies are nonlinear and are well described by $r=\sqrt{A}=r_{0}-\varepsilon .t^{2}$ , with ε = 0.0041 and 0.0031 for the second and third buried graphene layers, respectively. This behavior indicates that graphene etching proceeds in the supply-limited regime and that etching of the third layer is slower than that of the second layer. The etching rate increases with increasing oxygen pressure (Figure 3b), further confirming that etching of the buried layer proceeds in the supply-limited regime. Experiments performed at different temperatures reveal slower etching rates for buried layers compared with the overlayer (Figure 3c). Assuming Arrhenius-type behavior of the rate-limiting process, analysis of the temperature-dependent data (Figure 3c, inset) yields an activation energy *E*_A,O2_ = (3.48 ± 0.16) eV. Etching by hydrogen is also supply limited, as evidenced by fits to *r* = *r*_0_ – ε. *t* ^2^ (Figure 3d), and it exhibits thermally activated behavior (Figure 3e), yielding *E*_A,H2_ = (1.14 ± 0.24) eV. Etching by CO, however, exhibits distinct behavior. Fitting the areal etching kinetics (Figure 3f) with $r=\sqrt{A}=r_{0}-\varepsilon .t^{x}$ yields *x* = 1.58 ± 0.01, which lies between reaction-limited (*x* = 1) and supply-limited (*x* = 2) regimes. This indicates that buried-layer etching by CO is governed by multiple rate-limiting mechanisms. Moreover, the process does not follow Arrhenius-type behavior; instead, the etching rate decreases slightly with increasing temperature (Figure 3g). Thus, in contrast to O_2_- and H_2_-induced etching, CO-induced buried-layer etching is not thermally activated. As with other etchants, the etching of the buried layer is consistently slower than that of the overlayer.

**Figure 3:**
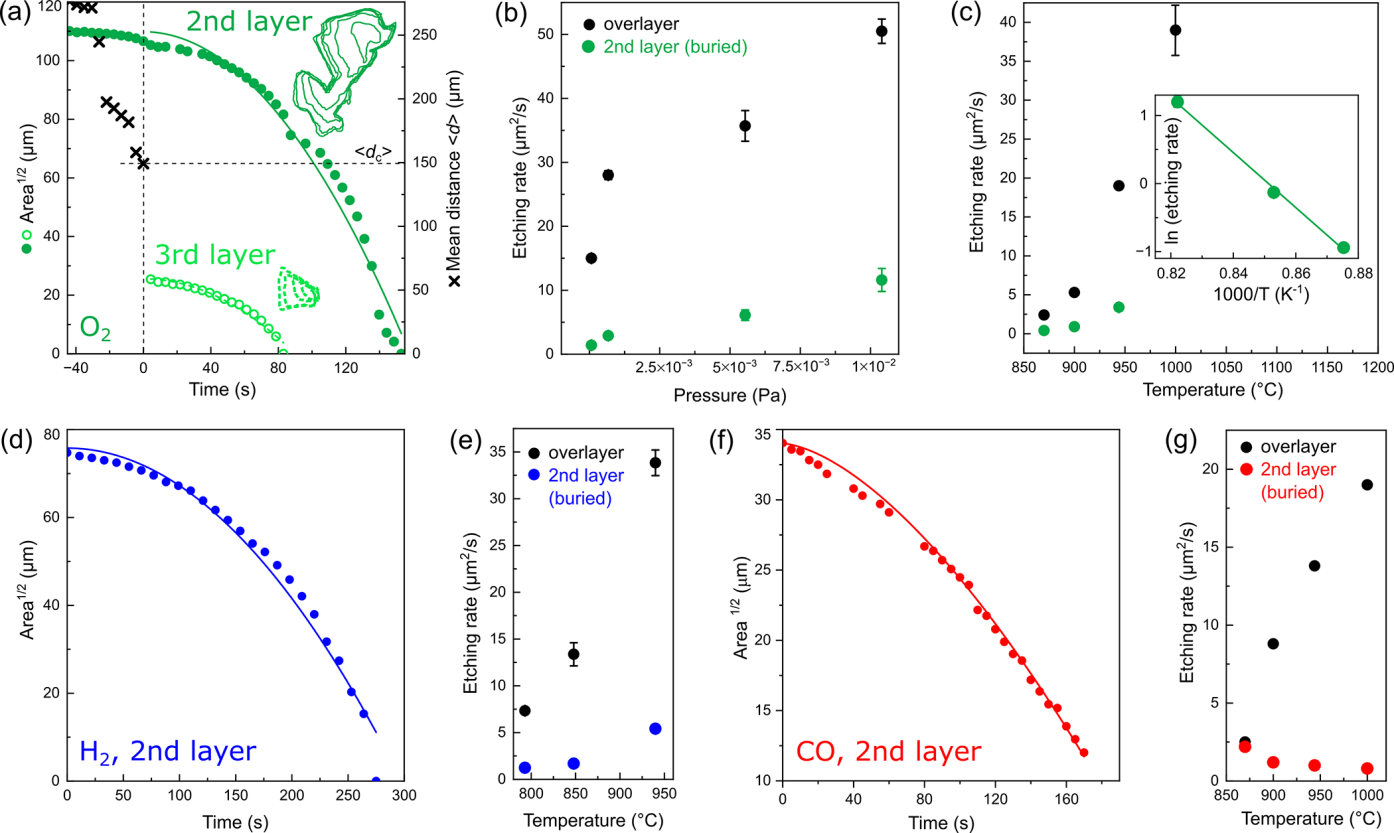
Etching kinetics of buried graphene layers in multilayer graphene. (a) Etching of the 2nd and 3rd graphene layers by oxygen, demonstrated by the time evolution of the square root of the flake area. *T* = 1000 °C, *p* = 6.6 × 10^–4^ Pa. The black crosses mark the mean distance between the overlayer etch front and the edge of the buried graphene flake. The mean critical distance (horizontal dashed line) marks the onset of etching at *t* = 0 s. (b) Dependence of the etching rate on oxygen pressure, *T* = 1000 °C. (c) Dependence of the etching rate on temperature (*p* = 3.0 × 10^–4^ Pa) for the overlayer and 2nd buried layer. The inset shows the same data in Arrhenius-type representation, allowing extraction of the activation energy. (d–g) Etching of the 2nd graphene layer by hydrogen (d, e) and CO (f, g). Experimental conditions: (d) *T* = 1000 °C, *p* = 5.0 × 10^–4^ Pa, (e) *p* = 7.3 × 10^–4^ Pa, (f) *T* = 1000 °C, *p* = 1.5 × 10^–3^ Pa. (g) *p* = 1.3 × 10^–3^ Pa.

Another quantity derived from the in situ observations (Figure 2) is the mean critical distance, ⟨*d*_c_⟩, which marks the onset of etching of the edge of the buried second graphene layer. We monitored the mean distance between the overlayer etch front and the edge of the buried second layer as they approached each other over time (see, for example, Figure 3a, crosses) and defined ⟨*d*_c_⟩ as the distance at which 3% of the buried-layer area had been etched. The mean critical distance thus reflects both the average transport distance of etchant species beneath graphene and their reactivity at the buried graphene edge. Figure 4a shows ⟨*d*_c_⟩ as a function of temperature for all three etchants. The data suggest a thermally activated mechanism; however, the large scatter prevents reliable determination of an activation barrier. This scatter is attributed to the polycrystalline nature of the Pt substrate.^58,59^ Notably, the ⟨*d*_c_⟩ values for CO are significantly larger than those for O_2_ and H_2_, indicating either a faster transport mechanism beneath graphene or an enhanced etching rate at the buried graphene edge for CO.

**Figure 4:**
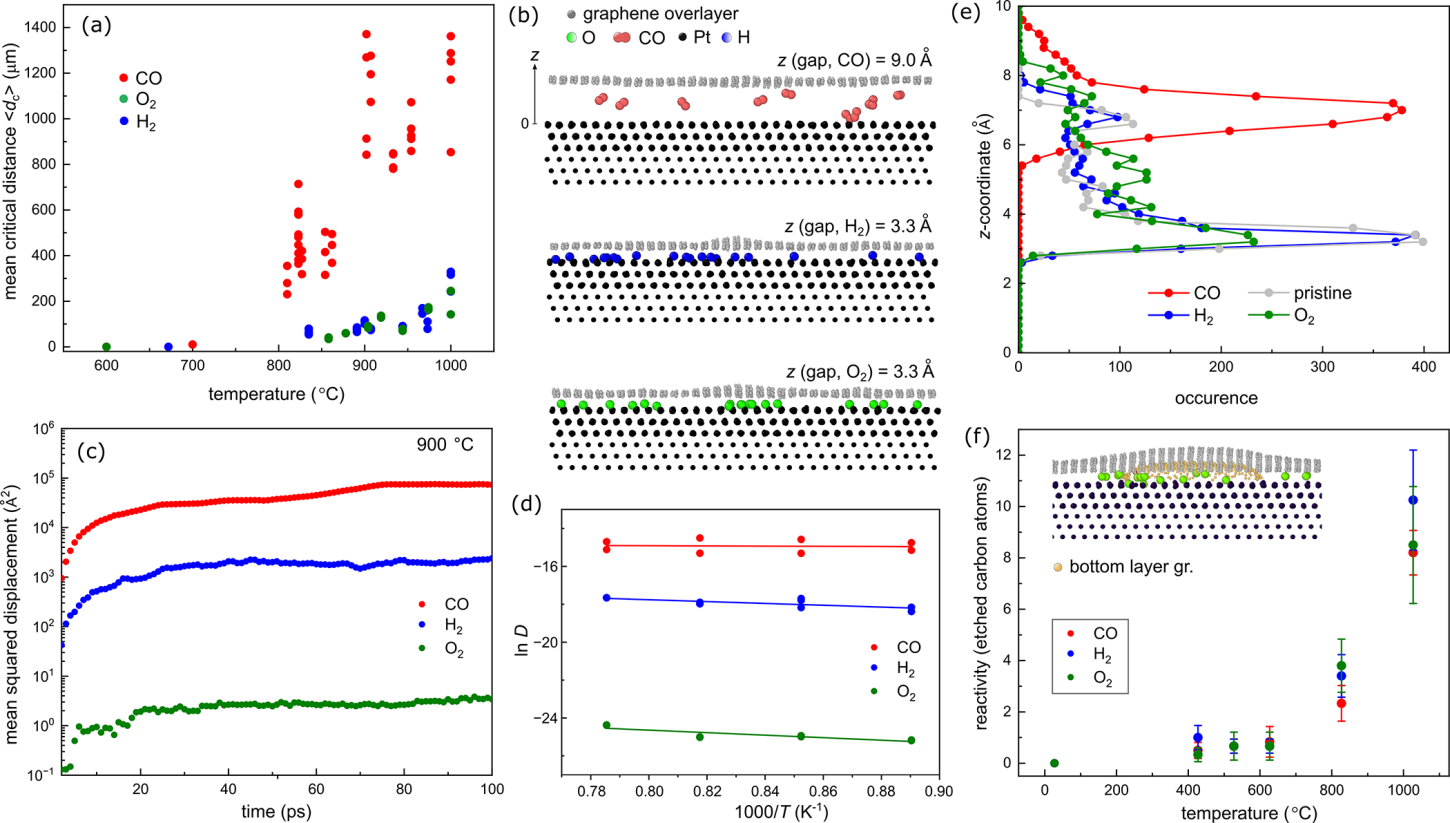
(a) Temperature dependence of the mean critical distance ⟨*d*_c_⟩ for graphene etching by O_2_, H_2_, and CO on polycrystalline Pt. The data were collected for pressures in a range of ⟨7.4 × 10^–4^ to 1.4 × 10^–3^ Pa⟩, see Figure S5 for details. (b) Snapshots from the molecular dynamics simulation of intercalated monolayer graphene at *T* = 900 °C (full movies available as Movies S1, S2, S3, Supporting Information). (c) Time-dependent mean squared displacement (MSD) of CO, H_2_, and O_2_ obtained from molecular dynamics simulations of diffusion within the vdW gap at *T* = 900 °C. (d) Arrhenius-type plots of the temperature dependence of the diffusion coefficient *D* derived from the simulations. (e) Z-coordinate (height) distributions of carbon atoms forming the top graphene layer for the relaxed bilayer graphene/Pt slabs (see inset in (f), calculated at *T* = 627 °C). The zero of the *z*-axis is set at the first Pt layer. (f) Temperature dependence of the reactivity of the buried graphene edge, expressed as the number of etched carbon atoms per edge atom for all three etchants. The inset shows a snapshot from a molecular dynamics simulation of bilayer graphene intercalated with oxygen at *T* = 627 °C. (note: (b–d) are simulations of intercalated monolayer graphene, and (e, f) are simulations of intercalated bilayer graphene.).

To gain further insight into the underlying mechanisms, we performed molecular dynamics simulations of the intercalated graphene/Pt system (Figure 4b). A key observation is that unlike oxygen or hydrogen, intercalated CO strongly decouples graphene from the Pt substrate, as evidenced by a pronounced increase in the van der Waals gap height (Figure 4c). This behavior is consistent with experimental observations from electron reflectivity measurements (Figure 1d).

We next analyzed the dynamics of intercalated molecules by characterizing their motion within a diffusive transport framework without a priori assuming a specific microscopic transport mechanism. Surface diffusion is commonly described as a random walk characterized by the mean squared displacement ⟨*x* ^2^⟩. In two dimensions, ⟨*x* ^2^⟩ = 4*D*τ, where *D* is the diffusion coefficient and τ is the adatom residence time on the surface. Diffusion is a thermally activated process, and the diffusion coefficient typically follows an Arrhenius-type dependence, $D=D_{0}\exp\left(-\frac{E_{A}}{k_{B}T}\right)$ , where *D*_0_ is the diffusion prefactor, *E*_A_ is the activation energy, *k*_B_ the Boltzmann constant, and *T* the temperature. By tracking individual atoms and molecules in the simulations, we extracted the time evolution of ⟨*x* ^2^⟩ (Figure 4c). The mean squared displacement of CO is significantly larger than those of hydrogen and oxygen. Activation energies derived from Arrhenius-type fits (Figure 4d) yield *E*_A,CO_ = (0.04 ± 0.20) eV, *E*_A,H2_ = (0.42 ± 0.13) eV, and *E*_A,O2_ = (0.57 ± 0.14) eV. The extremely low activation energy for CO suggests that CO can migrate almost freely beneath graphene, even at temperatures lower than those explored here. Consequently, *E*_A,CO_ likely does not correspond to a true diffusion barrier and rather relates to a different mechanism, as we discuss in Discussion.

Finally, we evaluated the etching dynamics at the buried graphene edge. The CO-intercalated bilayer graphene slab exhibits again a strong decoupling from the Pt substrate (Figure 4e). Yet, the reactivity, expressed as the number of etched carbon atoms per buried edge atom, does not differ significantly among the three etchants (Figure 4f). This observation supports the conclusion that the anomalous behavior of CO confined beneath graphene arises primarily from a distinct transport mechanism rather than enhanced edge reactivity.

In the case of CO-induced etching, our simulations indicate that, within the van der Waals gap, multiple graphene etching reaction pathways are plausible. We performed molecular dynamics simulations of dense CO gas confined between two platinum surfaces, with a graphene flake located on one of the Pt surfaces, at *T* = 1427 °C. This configuration enables a locally enhanced CO pressure near the Pt surface, representative of confinement within the van der Waals gap. Under these conditions, we observe additional reaction pathways that are absent on a pristine Pt surface at lower CO pressures. One such pathway involves carbon uptake by CO through a formation of a transient C_2_O intermediate (Figure 5a),^60^ which can subsequently lead to graphene etching and dissolution of carbon into the Pt substrate (C_2_O → CO + C (dissolved)). Another kinetically feasible pathway is the formation of CO_2_ from two CO molecules (Figure 5b), which may again be followed by graphene etching via the reverse reaction (C (graphene) + CO_2_ → 2CO).^61,62^ We note that these simulations were performed at elevated temperatures and locally high CO densities in order to accelerate low-frequency events within the time window accessible to reactive molecular dynamics. As such, the simulations shown in Figure 5 are intended to identify and characterize the plausible reaction pathways under confinement rather than to provide quantitatively accurate etching rates. While the simulation temperatures exceed experimentally accessible values, similarly high pressures within van der Waals gaps have been reported previously.^19^ Direct experimental verification of these confined reaction pathways nevertheless remains challenging due to the instrumental limitations of current in situ techniques, despite recent advancements in this field.^63^

**Figure 5:**
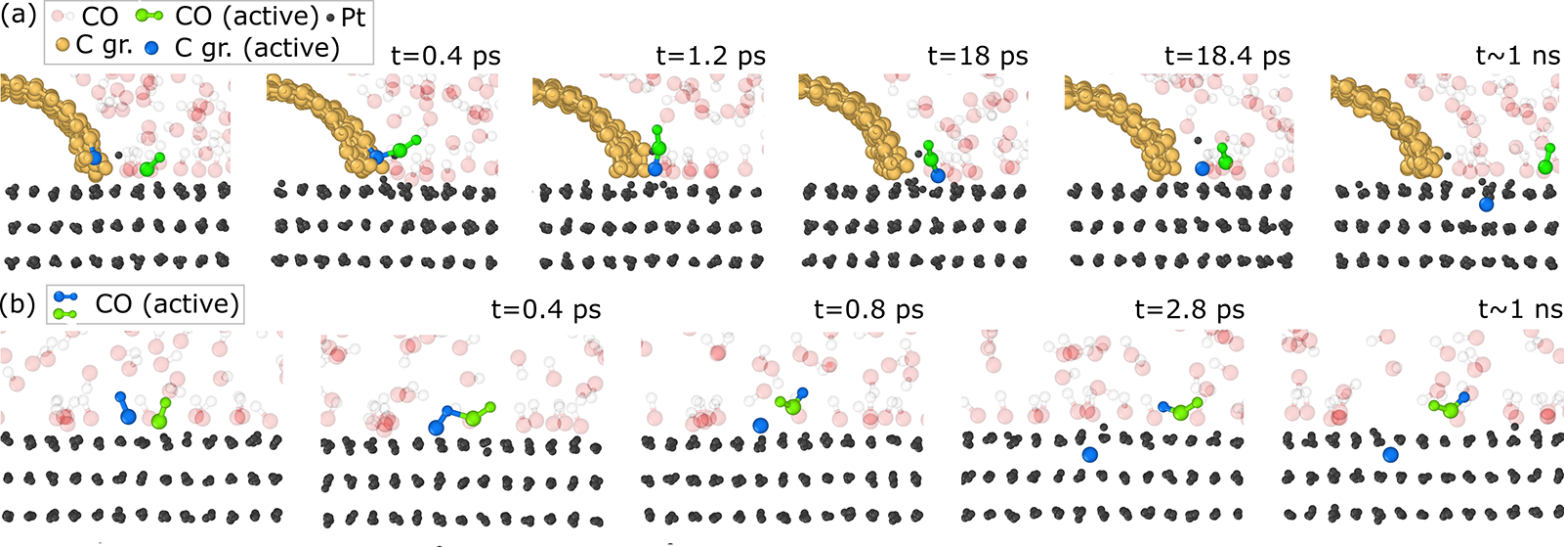
Molecular dynamics simulations of CO molecules confined between two Pt surfaces with a graphene layer supported on one of the Pt surfaces (the second Pt surface above is not visible in the images). (a) Formation of the C_2_O intermediate via carbon removal from graphene, CO + C (graphene) ↔ C_2_O ↔ CO + C (dissolved). (b) CO_2_ formation via (2CO → CO_2_ + C (dissolved). Gray: Pt atoms. Orange: graphene. Transparent red: C atoms in the CO molecules. Transparent white: O atoms in the CO molecules. Reactive atoms are highlighted in blue and green (in panel (a), blue indicates a graphene carbon atom and green indicates a CO molecule; in panel (b), blue and green indicate CO molecules). Large spheres represent carbon atoms, and small spheres represent oxygen atoms. The temperature is *T* = 1427 °C.

## Discussion

The LEED and LEEM data shown in Figure 1 are consistent with previous reports on several important aspects. Intercalation is possible even at room temperature through graphene edges, provided that the edges are terminated by adsorbed atoms or molecules.^13^ Otherwise, graphene edges remain strongly bound to the metal surface, effectively closing the vdW gap and preventing the intercalation. The requirement of edge termination as a prerequisite for intercalation was established previously. In the case of O_2_ and H_2_, molecular dissociation occurs readily upon adsorption on Pt, even at room temperature. Dissociation of both species is also observed in our molecular dynamics simulations (Figure 5b). A certain surface coverage by the atomic species is required before the graphene edges become terminated^13^ and lift from the metal surface, thereby opening the vdW gap for diffusing molecules and adatoms.^15^

It has been previously shown that, at temperatures below 600 °C, the decoupling of graphene edges from the metal surface constitutes the rate-limiting step for oxygen intercalation.^32^ Our data demonstrate that a supply-limited etching regime persists even at temperatures above 600 °C for both O_2_ and H_2_ (Figure 3). The activation energies extracted from the experimental data, *E*_A,O2_ = (3.48 ± 0.16) eV and *E*_A,H2_ = (1.14 ± 0.24) eV, are significantly higher than those of the corresponding diffusion barriers in the vdW gap obtained from molecular dynamics simulations. Consequently, these activation energies cannot be attributed to diffusion within the confined space but instead represent an effective energetic barrier associated with entry into the van der Waals gap. These findings indicate that, within the temperature range investigated here, and particularly for oxygen, the energetic cost of accessing the confined space plays a dominant role in the kinetics of intercalation.

Comparison of diffraction patterns (Figure 1) reveals that, at room temperature, adsorption sites on pristine Pt and under graphene remain identical (Figure 1).^44^ However, diffraction data alone do not provide conclusive evidence of molecular intercalation. In particular, the disappearance of the diffraction moiré pattern cannot be taken as definite proof of intercalation, as it may instead reflect an increase in the vdW gap height following intercalation. In the present work, gap enlargement is experimentally demonstrated for CO intercalation by electron reflectivity measurements (Figure 1d) and is further corroborated by molecular dynamics simulations (Figure 4b,e).

The increased vdW gap height also provides a natural explanation for the fast CO transport observed experimentally (Figure 4a) and in simulations (Figure 4c), as well as for the extremely low effective activation energy extracted for CO transport (∼0.04 eV). On free Pt surfaces, CO diffusion barriers are reported to lie between 0.39 and 0.56 eV.^59^ It has been discussed previously that the weaker confinement and a larger the vdW gap reduce the pressure exerted on the adsorbate molecule^19^ and decrease the strain imposed on graphene.^47^ Under such conditions, fast gas-phase transport within the vdW gap may become possible, bypassing slower surface diffusion of the adsorbed species. This scenario has been proposed previously^32,64^ and is strongly supported by our CO-related experimental observations. At elevated temperatures, we observe both the disappearance of adsorbate-induced diffraction patterns and a concurrent expansion of the vdW gap, suggesting that CO molecules remain confined within the gap predominantly in the gas phase rather than adsorbed at specific Pt sites. Molecular dynamic simulations further support this picture by revealing repeated desorption and readsorption events at the confining surfaces (Movie S1, Supporting Information). In contrast, the transport of oxygen and hydrogen within the vdW gap proceeds predominantly by surface diffusion. The diffusion barriers extracted from our simulations are comparable to literature values reported for free Pt surfaces (0.30–0.52 eV for hydrogen,^65^ and for oxygen, the literature data oscillate between 0.43–0.58 eV^58^ and 1.30–1.70 eV^59^), indicating that confinement does not significantly reduce the diffusion barriers for these species. Consequently, our data do not support a confinement-induced enhancement of diffusion for the two main compounds, O_2_ and H_2_.

The slower etching rates observed for the buried graphene layers (Figure 3) contrast with some recent reports, suggesting accelerated reaction kinetics under confinement.^40,50,63,66^ However, our findings are consistent with other studies in which no enhancement of buried-layer etching was observed.^12,67^ These results indicate that under the conditions studied here the supply-limited regime can outweigh any potential reduction in activation energies for reactions occurring within the vdW gap. A more systematic exploration of reaction kinetics under varied experimental conditions will be necessary to fully elucidate the role of confinement effects on vdW chemistry, including reactions beyond graphene etching, such as CO oxidation.

Two additional observations merit a discussion. First, CO has recently been identified as a promising precursor for graphene growth on copper,^68^ making the observation of graphene etching on platinum particularly surprising. Secondary ion mass spectroscopy depth profiling of Pt samples exposed to CO at etching temperatures reveals a significant amount of carbon dissolved within the Pt bulk, in contrast to pristine Pt samples not exposed to carbon-containing gases (Figure S6). This observation suggests that CO molecules may dissociate on the Pt surface at elevated temperatures, as proposed previously in ref 69 with subsequent dissolution of carbon into the Pt substrate. The remaining oxygen species would then be available to etch graphene. Why CO-induced graphene etching occurs only at higher pressures remains an open question.

Finally, the inability of etch reaction products to rapidly escape from the confined vdW gap leads to unexpected phenomena. One example is the transient growth of graphene on the side of a flake opposite the etch front during oxygen-induced etching. In the absence of efficient escape pathways, reaction products such as CO may accumulate within the vdW gap, dissociate, and locally supersaturate graphene edges with carbon, thereby initiating growth. The asymmetric flow of etchant species in our experiments dictates that such growth occurs preferentially at the edges farthest from the advancing graphene overlayer etch front. As the etch front progresses and the local concentration of etchant increases, this growth is rapidly reversed, consistent with previous observations.^50^ This growth behavior cannot be attributed to Ostwald ripening, as graphene flakes remain stable under a vacuum at elevated temperatures. Accumulation of reaction products may affect etching kinetics in multilayer graphene systems, as evidenced by deviations from ideal fits in trilayer etching data (Figure 3a). Under certain conditions, reaction products may block adsorption sites or otherwise restrict the supply of reactants to the reaction front within the vdW gap.

## Conclusions

In summary, we have employed in situ electron microscopy to directly monitor graphene etching reactions occurring within the van der Waals gap between a platinum substrate and CVD-grown graphene. Our results demonstrate that, for all three etchants studied (O_2_, H_2_, and CO), the etching of buried graphene layers proceeds in a supply-limited regime within the accessible experimental range (up to 1.35 × 10^–2^ Pa). In all cases, etching within the van der Waals gap is slower than etching of the exposed graphene overlayer, highlighting the entrance to the vdW gap and mass transport as the dominant kinetic constraints under confinement.

Importantly, our combined experimental and molecular dynamics results reveal that intercalation does not simply reduce activation barriers for surface reactions. Instead, confinement within the van der Waals gap fundamentally alters the reaction environment by modifying adsorption states and enabling reaction pathways that are absent on open surfaces. This effect is particularly evident for CO, where gap expansion, gas-phase-like transport, and additional carbon-transfer reactions emerge under confinement, effectively mimicking high-pressure reaction conditions at comparatively low external pressures.

These findings establish van der Waals gaps as chemically distinct nanoreactors in which reactivity is governed not only by energetic barriers but also by confinement-induced mass-transport effects. Future efforts to exploit confined spaces, for e.g., catalysis, should therefore focus on engineering mass transport—through control of gap height, choice of two-dimensional cover layers, or substrate chemistry—rather than relying solely on barrier lowering. More broadly, our work suggests that intercalation under two-dimensional materials offers a powerful platform for studying high-pressure and confinement-driven chemistry using conventional surface-science and analytical techniques.

## Materials and Methods

The initial experiments shown in Figure 1 were carried out in an ultrahigh vacuum (UHV) system (base pressure ∼10^–8^ Pa), which houses a SPECS FE-LEEM P90 low-energy electron microscope and SPECS X-ray photoelectron spectroscope (XPS) with the Mg Kα X-ray source and Phoibos 150 spectrometer, among other tools, and allows transfer of samples in vacuo under UHV conditions. A single crystal sample of Pt(111) (SPL, The Netherlands) was annealed at 905 °C in an oxygen atmosphere (1.3 × 10^–4^ Pa) to remove all residual contamination. The sample cleanliness was checked by LEED, XPS, and bright-field LEEM imaging. Graphene was grown in a preparation chamber by exposing the clean Pt sample to ethylene gas (Ethylene 3.5, Messer) at 950 °C (5–7 × 10^–6^ Pa) until the desired coverage was reached. Molecular intercalation was performed directly in the LEEM chamber by introducing the relevant gas (Oxygen 5.0 (Messer), Hydrogen 5.0 (Messer), and CO 3.7 (Linde)) with a leak valve. The intercalation pressures were 1.6 × 10^–4^ Pa.

The sample utilized for all etching experiments was a Pt wire (0.2 mm or 0.25 mm diameter, Goodfellow, 99.99% purity), which was hammered to expand the planar surface for graphene growth. Subsequently, the sample was annealed at an elevated temperature (>1000 °C) in the presence of oxygen inside an ultrahigh vacuum chamber equipped with a scanning electron microscope (Tescan). The base pressure in the main chamber was 4.8 × 10^–7^ Pa. The sample annealing was done by direct current resistive heating until all detectable carbon residues were removed and a clean surface with well-defined grains was achieved. Temperature was measured with an infrared pyrometer Micro-Epsilon CT-M3 H1-SF at a wavelength of 2.3 μm. The emissivity was set to 0.1. To grow graphene, ethylene gas (Messer, Ethylene 3.5) was supplied to the microscope chamber by a leak valve. First, we grew a monolayer of graphene through ethylene decomposition at 1–2 × 10^–4^ Pa at elevated temperatures (in the range of 900–1000 °C). Then, to form an inverted wedding cake configuration, we dissolved the graphene in the platinum substrate by increasing the substrate temperature by 100 °C with respect to the growth temperature of the first graphene layer. Subsequently, we slowly decreased the temperature, which resulted in carbon segregation and graphene flake formation on the Pt surface. In addition to single-layer graphene flakes, carbon segregation leads to formation of additional graphene layers below the already formed graphene flakes (see Figure S7 for SEM images taken during the growth process). Such a multilayer configuration is called an inverted wedding cake, and the number of layers can be counted from the contrast in the secondary electron image. To perform etching, the etching agents were introduced into the chamber by additional leak valves.

Scanning electron microscopy observation during the etching reaction was performed by using a 5 keV electron beam with a current of 3 nA. Secondary electron images were formed by means of the Everhart–Thornley detector inside the main chamber.

The etching kinetics of buried graphene layers and the mean critical distance were quantified by SEM image analysis using ImageJ, Python-based routines, or a combination of both, depending on the image quality under different temperatures and atmospheres. Segmentation was performed using the Trainable Weka Segmentation plugin, and the temporal evolution of segmented areas was quantified using the Analyze Particles function for each frame. Python-based image processing followed a similar workflow with enhanced flexibility to account for the variable image quality. After region of interest selection, images were denoised using Gaussian filtering, contrast-enhanced by intensity inversion, and segmented using Otsu thresholding. Morphological operations were applied to refine the segmented features, after which contours were identified and converted to real units to quantify the area of the segmented regions as a function of time.

In situ XPS analysis was performed without charge neutralization. Survey spectra were collected in high-magnification mode using a pass energy of 100 eV with a dwell time of 100 ms and a 1 eV energy step. Detailed spectra were acquired in high-magnification mode using a pass energy of 25 eV with a 500 ms dwell time and 0.1 eV energy step, integrating 20–30 sweeps. All of the spectra were collected in a normal emission geometry (emission angle parallel to the surface normal). No spectral shifts were performed. The spectra were further processed using Casa XPS software. The C 1s, O 1s, and Pt 4p_3/2_ peaks (Figure S1) were fitted using the Shirley background and several components with parameters described in the main text and in Table 1 below: BE, binding energy; shift, with respect to the first component; fwhm, full width at half-maximum.

**Table 1:** Summary of XPS Peak Fitting Parameters

peak	assignment	shape	BE (eV)	shift (eV)	fwhm (eV)
C 1s	graphene	asymmetric Voigt: LA(1.53,243)	283.9		1.06
C 1s	graphene intercalated	asymmetric Voigt: LA(1.53,243)	283.8	0.1	1.10
C 1s	CO on Pt (111)	asymmetric Voigt: LA(1.53,243)	286.4		1.87
C 1s	CO beneath graphene	asymmetric Voigt: LA(1.53,243)	286.2	0.2	2.05
O 1s	CO on Pt (111)	asymmetric Voigt: LA(1.45,150)	532.08		3.47
O 1s	CO beneath graphene	asymmetric Voigt: LA(1.45,150)	531.71	0.37	2.60
Pt 4p_3/2_	Pt + ads. CO	asymmetric Voigt: LA(2.03,4.7,0)	519.23		5.01
Pt 4p_3/2_	Pt + graphene	asymmetric Voigt: LA(2.03,4.7,0)	518.95	0.28	4.53
Pt 4p_3/2_	Pt + CO-intercalated graphene	asymmetric Voigt: LA(2.03,4.7,0)	519.15	0.08	4.78

Depth profiling of platinum samples was performed using secondary ion mass spectrometry (SIMS) to elucidate subsurface compositional variations of clean and gas- and temperature-treated Pt samples. All analyses were conducted by using a TOF-SIMS5 instrument (IONTOF GmbH, Germany), allowing for high-resolution depth profiling at the nanometer scale. Bi^+^ primary ions were employed under the following conditions: an impact energy of 30 keV, an incidence angle of 45°, a pulsed primary current of approximately 3 pA, and raster dimensions of 100 μm × 100 μm^2^. To enhance the depth resolution and compensate for matrix effects, Cs^+^ (2 keV, 130 nA) cosputtering was used in noninterlaced sputtering mode. The two beams (sputtering and analysis) were alternating as follows: two Cs^+^ sputtering frames were followed by a 0.1s pause and by four Bi^+^ analysis frames. Measurements were carried out in negative polarity mode with an electron flood gun to compensate for surface charging during sputtering. The resulting depth profiles were plotted as the signal intensity versus sputter time. These profiles facilitated a qualitative relative comparison of carbon distribution across differently treated platinum samples.

Classical molecular dynamic simulations were performed using the LAMMPS simulation package.^70^ The reaxFF_C/H/O/Pt_ interatomic potential^71^ was adopted, which has been proven capable of describing complex chemical processes and reasonably accurate in modeling the interactions between carbon (C), hydrogen (H), oxygen (O), and platinum (Pt) atoms. Each MD configuration of the intercalated graphene/Pt system consisted of a single-crystalline Pt (111) substrate with a thickness of 1.2 nm and either one (overlayer) or two layers of graphene (overlayer and buried layer) above in an inverse wedding cake configuration. In each system, 10 CO, H_2_, or O_2_ gas molecules were randomly added in the region between the graphene and the substrate, and above the graphene layers, a vacuum region was created with a thickness of around 5 nm. The *x* and *y* dimensions of the simulation boxes were maintained at 9.00 and 7.80 nm, respectively, under periodic boundary conditions, as shown in Figure S8. For simplicity, the thermal expansion of the substrate is not accounted for (and found to have marginal effects on molecule diffusion). A Nose–Hoover thermostat^72^ was employed to control the temperature. The velocity Verlet algorithm^73^ with a time step Δt = 0.2 fs was applied, and the total length of each simulation was greater than 3 ns. For each condition investigated in this study, MD runs were repeated five times independently, and the coordinates of the atoms were recorded every 1 ps for postprocessing analyses.

## Supplementary Material









## Data Availability

The data underlying this study are openly available at 10.5281/zenodo.15849416.

## Notes

XPS analysis of CO-adsorbed Pt and CO-intercalated graphene/Pt stack; electron reflectivity of CO-intercalated graphene at elevated temperatures; stability of graphene at low CO pressures; the electron beam effect evaluation; the mean distance dependence on temperature; the SIMS depth profile of platinum exposed to CO; the SEM sequence demonstrating the wedding cake graphene formation; and the initial MD configuration of the intercalated monolayer graphene/Pt system (PDF)
Molecular dynamics simulation snapshots of CO confined in the vdW gap beneath monolayer graphene on Pt at *T* = 900 °C (MP4)
Molecular dynamics simulation snapshots of O_2_ confined in the vdW gap beneath monolayer graphene on Pt at *T* = 900 °C (MP4)
Molecular dynamics simulation snapshots of H_2_ confined in the vdW gap beneathmonolayer graphene on Pt at *T* = 900 °C (MP4)
