# Supplementary material for: Confined Reactivity in the van der Waals Gap beneath Graphene: Supply-Limited Kinetics and Emergent Reaction Pathways
Source: ACS Nano. 2026 Mar 4;20(10):8255–65. doi: 10.1021/acsnano.5c12130 (PMC13001084; doi:10.1021/acsnano.5c12130)
Supplement: Supplementary file 1 [file nn5c12130_si_001.pdf]

Supporting Information for

Confined Reactivity in the van der Waals gap beneath graphene: Supply-Limited Kinetics and Emergent Reaction Pathways

Hossein Mirdamadi[1#], Rui Wang[2#], Jiří David[1,3#], Tianle Jiang[4], Yanming Wang[5], Karel Vařeka[1,3], Michal Dymáček[3], Petr Bábor[1,3], Tomáš Šikola[1,3], Miroslav Kolíbal[1,3*]

[1]CEITEC BUT, Brno University of Technology, Purkyňova 123, 612 00 Brno, Czech Republic

[2]School of Mechanical Engineering, Shanghai Jiao Tong University, 800 Dongchuan Rd, Shanghai, China, 200240

[3]Institute of Physical Engineering, Brno University of Technology, Technická 2, 616 69 Brno, Czech Republic

[4]University of Michigan-Shanghai Jiao Tong University Joint Institute, Shanghai Jiao Tong University, 800 Dongchuan Rd, Shanghai, China, 200240

[5]Global Institute of Future Technology, Shanghai Jiao Tong University, 800 Dongchuan Rd, Shanghai, China, 200240

[#]contributed equally to this work

[*]kolibal.m@fme.vutbr.cz

Contains supporting figures S1-S8 and description of supporting movies S1-S3.

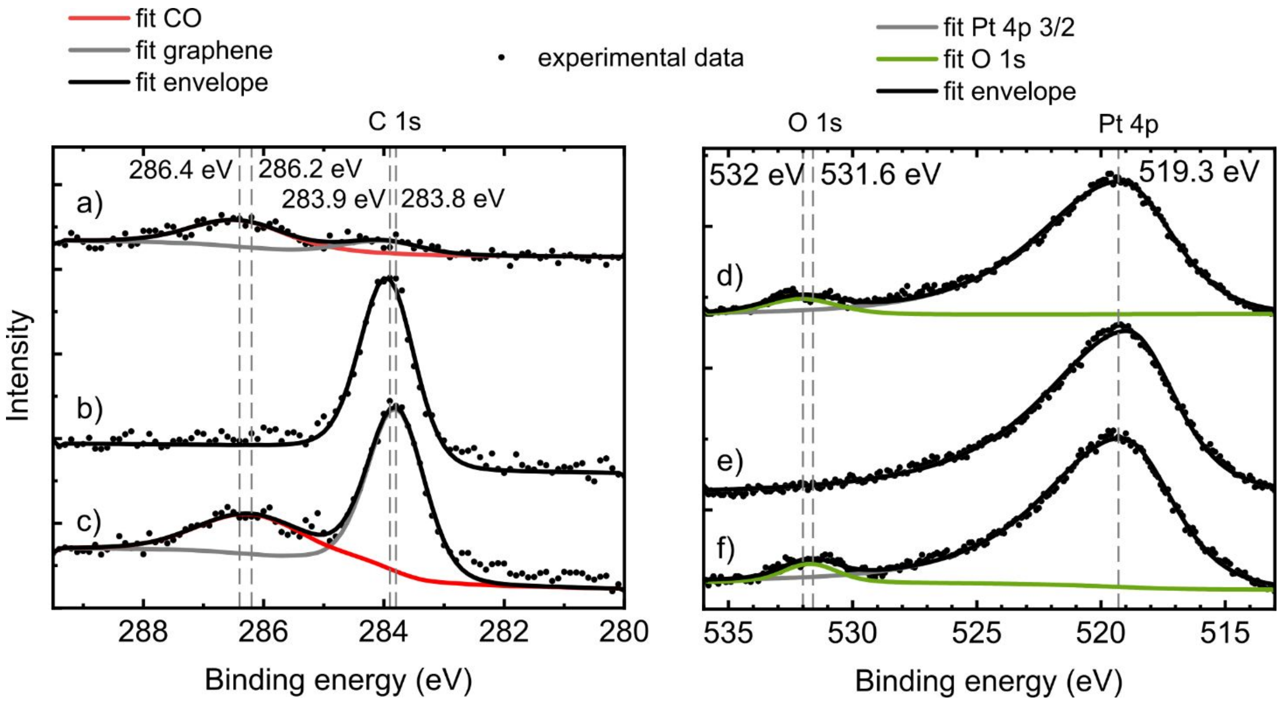

Fig. S1: XPS analysis of (a,d) CO-adsorbed Pt, (b,e) non-intercalated graphene/Pt and (c,f) CO-intercalated graphene/Pt. Left: C 1s region, right: O 1s region. See caption of Fig. 1 for additional experimental details.

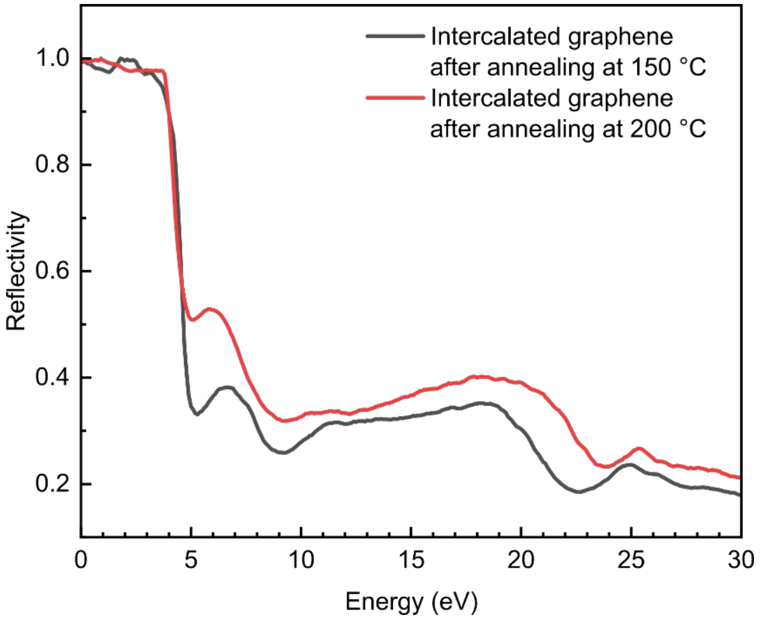

Fig. S2: Electron reflectivity of CO-intercalated graphene at elevated temperatures. The two minima between 5 and 10 eV persist even at elevated temperatures.

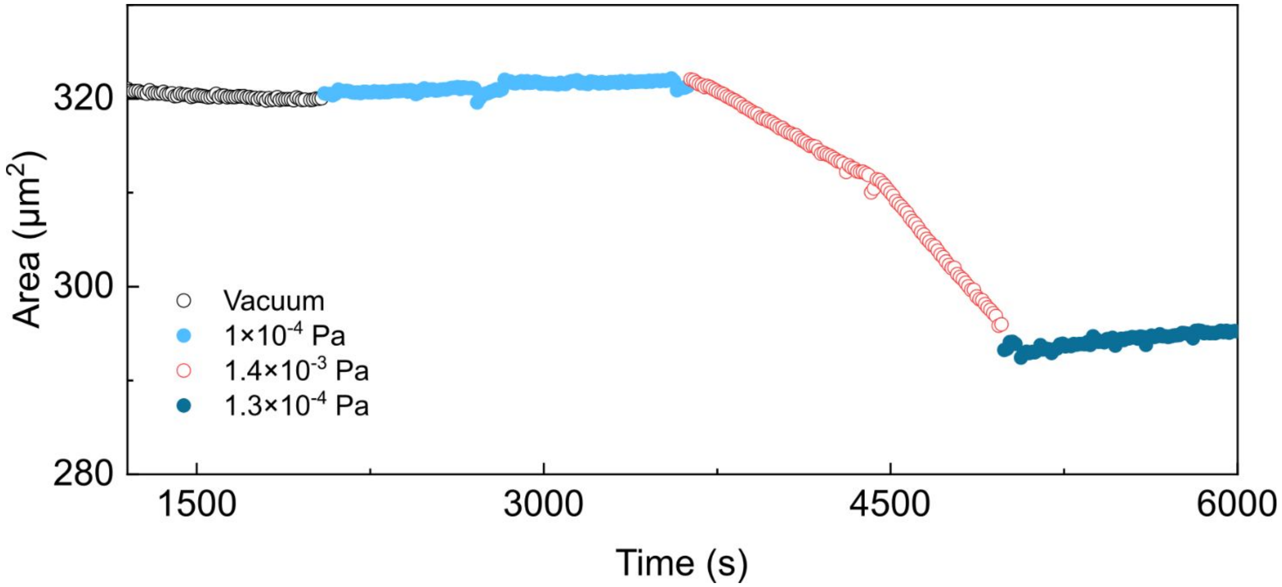

Fig. S3: Stability of graphene at low CO pressures. The plot shows time-dependence of the graphene flake area (as calculated from SEM images) at 834 °C at different CO pressures. At low partial CO pressures (low 10^{-4} Pa range) the flake area slightly increases, possibly due to the Boudouard reaction, $2CO \leftrightarrow C+CO_2$, similarly to the other reports [55]. If the CO pressure is increased up to 10^{-3} Pa range, the flake behaviour is reversed and instead of growth we observe a rapid etching of the flake.

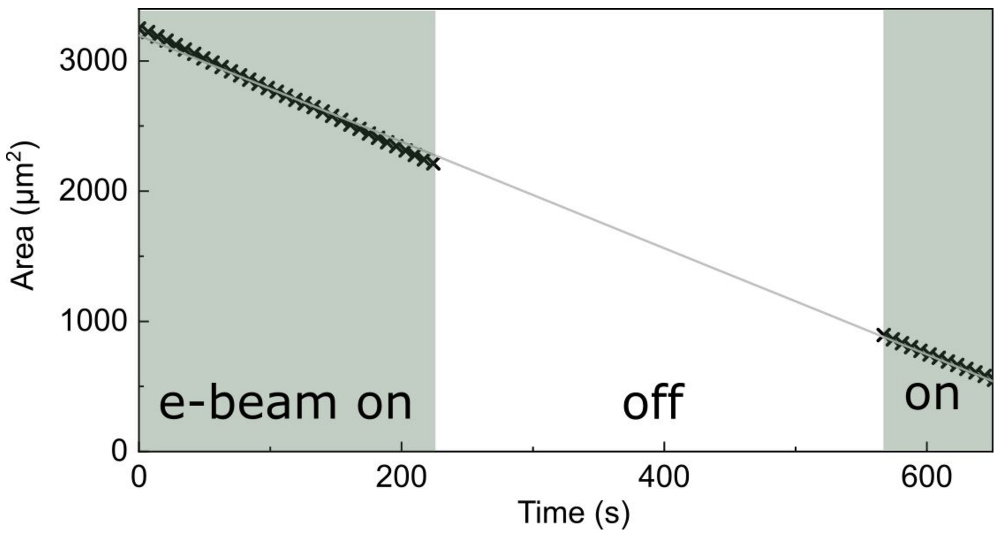

Fig. S4: The effect of the electron beam exposure (5 keV, 300 pA) on the observed graphene area reduction during etching by oxygen gas (T = 1000 °C, p = 1.6 × 10^{-6} Pa). The deviation from the grey line (which represents a constant etch rate) is negligible.

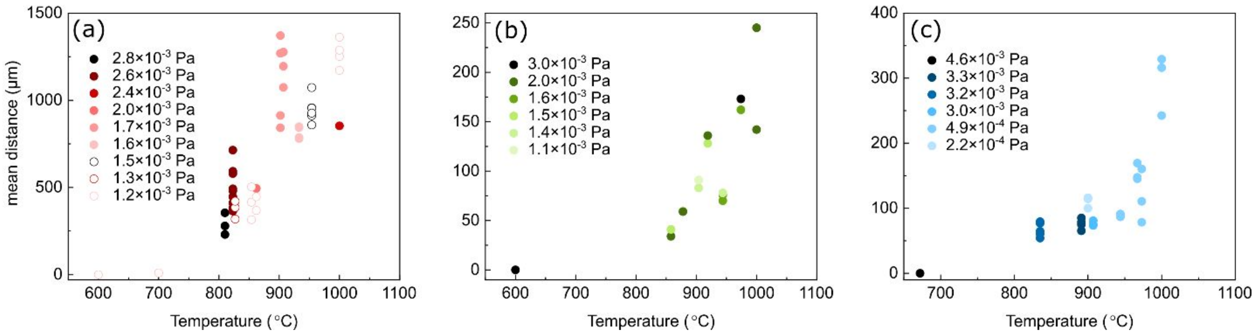

Fig. S5: The mean distance (between the overlayer etch front and the edge of buried 2nd layer graphene) dependence on temperature, as measured for distinct pressures (as marked in the legends) and different gases: (a) CO, (b) O_2 (c) H_2.

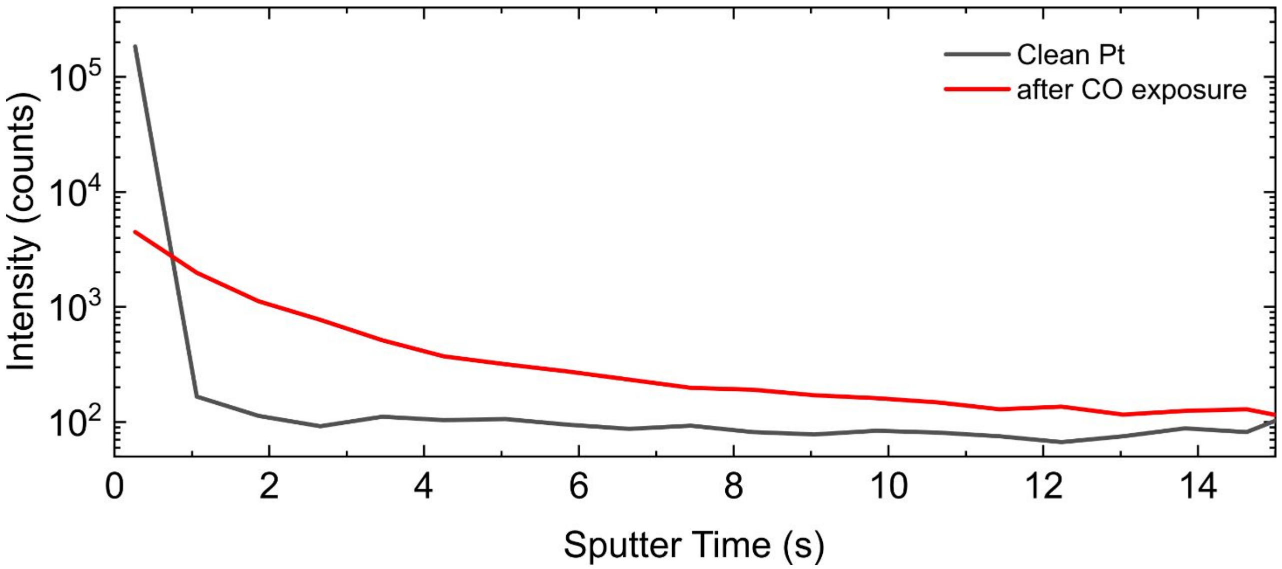

Fig. S6: SIMS depth profile (C^- signal) of platinum exposed to CO. Grey data: depth profile of a clean platinum surface (annealed in oxygen, 1.2×10^{-4} Pa, for 10 minutes at temperature slightly above 1000 °C). Red data: depth profile of the same sample after exposure to CO (1.8×10^{-3} Pa for 40 minutes at temperature slightly above 1000 °C. We first evacuated all the CO from the chamber and only then cooled down the sample to room temperature, to avoid false carbon-related signal detection, potentially due to adsorbed CO molecules.

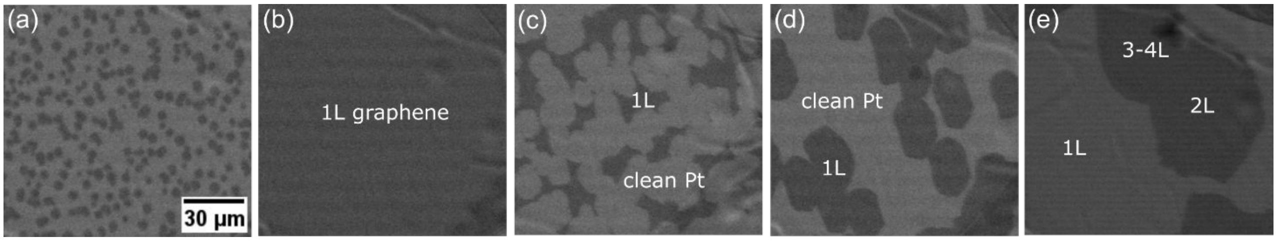

Fig. S7: SEM sequence demonstrating the wedding cake graphene formation. (a,b) A full graphene overlayer (1L) is created on Pt at 1000 °C by exposure to ethylene. Then, the ethylene flow is stopped. (c) As the temperature is increased to 1200 °C, graphene gets dissolved in the Pt substrate. (d) By decreasing the temperature below 1000 °C, the dissolved carbon segregates at the Pt surface and forms graphene overlayer. (e) Subsequently, multilayer graphene is formed by additional carbon segregation below the graphene overlayer. The number of layers is counted based on the contrast in the secondary electron image.

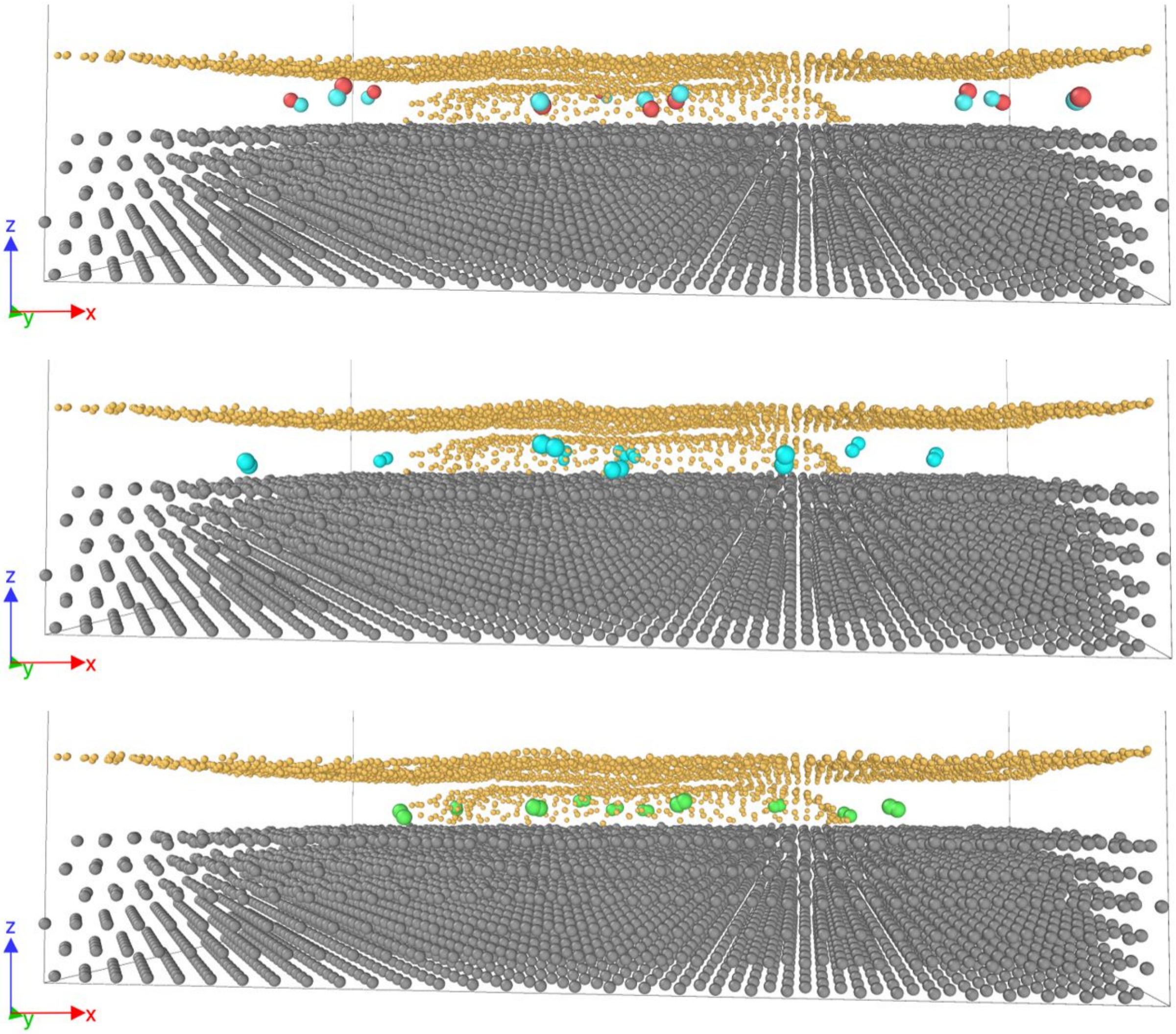

Fig. S8: Initial MD configuration of the intercalated bilayer graphene/Pt system. Gray: Pt atoms. Orange: graphene. Red: C atom in CO molecules. Blue: O atoms. Green: H atoms.

Movie S1. Molecular dynamics simulation snapshots of CO confined in the vdW gap beneath monolayer graphene on Pt at *T* = 900 °C. The trajectory shown spans 1–2 ns, with frames saved every 20 ps.

Movie S2. Molecular dynamics simulation snapshots of O_2 confined in the vdW gap beneath monolayer graphene on Pt at *T* = 900 °C. The trajectory shown spans 1–2 ns, with frames saved every 20 ps.

Movie S3. Molecular dynamics simulation snapshots of H_2 confined in the vdW gap beneath monolayer graphene on Pt at *T* = 900 °C. The trajectory shown spans 1–2 ns, with frames saved every 20 ps.
